# Body composition as a predictor of cancer-related death in colon cancer: an AI-based volumetric analysis

**DOI:** 10.1007/s11547-026-02232-x

**Published:** 2026-06-20

**Authors:** Michela Polici, Benedetta Masci, Damiano Caruso, Stefano Nardacci, Lapo Nardoni, Fiammetta Pacelli, Marta Zerunian, Domenico De Santis, Paolo Mercantini, Enrico Fiori, Mariarita Tarallo, Maria Ciolina, Riccardo Ferrari, Sang Joon Park, Jong-Min Kim, Marco Francone, Andrea Laghi

**Affiliations:** 1 Department of Medical-Surgical Sciences and Translational Medicine, School of Medicine and Psychology, Sapienza University of Rome – Sant’Andrea University Hospital, Via Di Grottarossa, 1035-1039, 00189 Rome, Italy; 2 School in Traslational Medicine and Oncology, Department of Medical-Surgical Sciences and Translational Medicine, Faculty of Medicine and Psychology, Sapienza University of Rome, Rome, Italy; 3 Department of Surgery, Policlinico Umberto I, Sapienza University of Rome, Via Giovanni Maria Lancisi, 2, 00161 Rome, Italy; 4 Department of Emergency Radiology, Azienda Ospedaliera S. Camillo-Forlanini, Rome, Italy; 5 Department of Radiology, Seoul National University College of Medicine, Seoul, Republic of Korea; 6 AI Center, Medical IP, Co., Ltd., Seoul, Republic of Korea; 7 Department of Biomedical Sciences, Humanitas University, Via Rita Levi Montalcini 4, 20072 Pieve Emanuele, Milan Italy; 8 Department of Diagnostic Imaging, IRCCS Humanitas Research Hospital, Via Manzoni 56, 20089 Rozzano, Milan Italy

**Keywords:** Colon cancer, Body composition, Prognosis, Cancer-related death

## Abstract

**Purpose:**

To investigate body composition as a predictive biomarker of cancer-related death in patients with non-metastatic colon cancer (CRC).

**Materials and methods:**

Patients with CRC (stages II–III) treated with upfront surgery, with availability of baseline CT, clinico-histological and survival data were retrospectively enrolled. Patients with stage IV or CT unavailability were excluded. Body composition parameters derived from baseline abdominal CT using AI-based automatic segmentation software. Seventy-six parameters regarding adipose visceral fat(AVF), subcutaneous fat(SF), bone density, liver density, and fat-fraction were automatically extracted from both whole segmentation volume and multislices region. According to the CRC-related death (CRC-related death), the population was divided into Group 1 (CRC-related death) and Group 2 (non-CRC-related death). Body composition features were compared between two groups. Predictive model and survival analysis were performed with ROC curves, Cox regression, and Kaplan–Meier method. *P* < 0.05 was considered significant.

**Results:**

A total of 293 patients were included, 101/293(34.5%) with CRC-related deaths. MeanHU of AVF and SF resulted directly correlated with CRC-related death (*P* = 0.004 and HR = 1.03, *P* = 0.002 and HR = 1.04, respectively) for the multislice analysis. MeanHU of AVF resulted in direct correlation with CRC-related deaths, also for the volumetric analysis (*P* = 0.04 and HR = 1.02). In multivariable Cox regression analysis, MeanHU AVF was confirmed as an independent predictor of CRC-related death in both multislice and volumetric analyses. SF HU remained significant in multislice analysis. In Kaplan–Meier analysis, AVF and SF for the multislice analysis resulted statistically significant (*P* = 0.033 and < 0.001, Chi-square = 4.56 and 11.7, respectively).

**Conclusion:**

In conclusion, our study demonstrated that body composition metrics of visceral and subcutaneous fat were significantly associated with cancer-related death in non-metastatic CRC patients.

## Introduction

Colon cancer is one of the most common cancers worldwide, and the patient’s prognosis is mostly dependent on clinical staging, cancer behavior, histology, and genetic paneling [1–3]. These aspects influence the choice between upfront surgery, eventually followed by adjuvant chemotherapy, and a neoadjuvant approach [2, 4]. Nevertheless, not all patients respond to treatment in the same way due to multiple aspects, the most common of which are clinical T and N staging in the preoperative CT scan [5], the different aggressiveness of the neoplasms, or the different body composition or nutrition status of the patient [6–8]. Numerous studies have highlighted the weaknesses of colon cancer workup [6–8], and multiple solutions are being proposed to move the patient toward a more personalized approach [5].

Regarding body composition and nutrition, the field of research is still open and at times undefined. As of today, the role of nutrition and changes in body composition has been studied in several cancer patient populations, acquiring a prognostic role in terms of survival and surgical outcome. In fact, the presence of sarcopenia has been shown to be one of the major body composition biomarkers in terms of worse survival, surgical complications, and high therapy-related toxicity in numerous cancers [9, 10]. However, minimizing body composition in the simple study of sarcopenia is reductive; in fact, a high number of other parameters can be extracted, with particular focus on the analysis of adipose visceral fat (AVF) and subcutaneous fat (SF) [9, 11].

CT imaging allows the assessment of both AVF and SF quality, as density is inversely correlated with adipocyte size. Lower density values indicate the presence of hypertrophic adipocytes, while low density has been associated with a high risk of cardiometabolic diseases [11]; additionally, high density has emerged as a negative prognostic factor in elderly and cancer patients [12].

Up to now, two main segmentation methods have been used: manual 2D segmentation at the level of the third lumbar vertebra (L3) and automatic 3D segmentation of the entire abdomen by using an AI-based software. The volumetric segmentation extracts multiple body composition parameters and overcomes the known limitations of 2D manual segmentation, such as the time-consuming approach, the risk of low reproducibility, and the reduced image sampling. To the best of our knowledge, there are no studies investigating the role of body composition and nutritional status as survival biomarkers in non-metastatic colon cancer (stages II and III), by analyzing the preoperative CT scans with an Artificial Intelligence (AI)-based software able to provide a volumetric analysis.

The aim of this study was to investigate whether body composition parameters might be integrated into the clinical workflow of colon cancer non-metastatic patients as survival biomarkers, in terms of cancer-related death, by extracting the volumetric measurements from the preoperative CT using an AI-driven software.

## Materials and methods

### Study population

This retrospective analysis was conducted within the framework of a 5-year multicenter trial, funded by AIRC IG 2020—ID 24974, approved by the ethical committee of Sant’Andrea University Hospital (ref. nr. CE 6597/2021), registered on ClinicalTrials.gov (NCT06108310).

The initial population of 557 individuals was screened from January 2015 to December 2021, all patients were affected by colon cancer at stages II–III, according to the AJCC 8th edition [13]. Patients were enrolled from two centers, Sant’Andrea and Policlinico Umberto I, Sapienza University Hospitals in Rome. All patients provided informed consent. Inclusion criteria required were the following: (I) availability of preoperative baseline CT images of the abdomen, (II) availability of clinical and histopathological data, and (III) upfront radical surgery. Patients excluded from the study met one of the following exclusion criteria: (I) metastatic disease, (II) treatment with neoadjuvant therapies, (III) any other concomitant or previous malignancies, and (IV) unavailability of preoperative CT images.

### Clinical data

For all patients, clinical data such as sex, age, adjuvant chemotherapy, and histopathological data such as T, N, extramural vascular invasion (EMVI), and tumor histology were collected. For all patients, weight and height were also collected. Furthermore, patients were also followed after surgery for 36 months to assess progressive disease, overall survival, and the occurrence of cancer-related death (CRC-related death). Patient cancer-related death was collected through the Hospital death registry. Based on mortality data, the population was divided into Group 1 (CRC-related death) and Group 2 (non-CRC-related death).

### CT images acquisition

All patients underwent contrast-enhanced CT at the time of staging on a 128-slice CT scanner (GE Revolution EVO CT Scanner, GE Medical Systems), in the supine position, with full inspiration and cranio-caudal direction, with the following CT specifications: tube voltage: 100 kVp, using an automatic current modulation range 100–240 mA (Auto-mAs, GE Healthcare), detector collimator configuration with z-flying focal spot technique: 0.625 × 64 mm; pitch: 0.984:1; gantry speed: 0.6 s; and beam collimation: 40mm.

High-concentration iodinated contrast medium (Iomeprol 400 mgI/mL, Iomeron 400; Bracco Imaging) was injected intravenously via an 18-gauge antecubital access at a flow rate of 3 mL/s. The volume of contrast medium injected was calculated by lean body weight using the James formula [14], followed by a 50 mL saline flush. The scan delay was determined using the bolus tracking technique (SmartPrep, GE Healthcare) by placing a region of interest (ROI) with a threshold of 100 Hounsfield unit (HU) within the abdominal aorta. The examinations included the unenhanced and portal venous (70 s from the threshold). The same CT protocol was also applied to the subsequent follow-up CT scans. This was a multicentric study in terms of enrollment; however, all CT acquisitions were centralized at Sant’Andrea University Hospital.

### CT images analysis

**Fig. 1:**
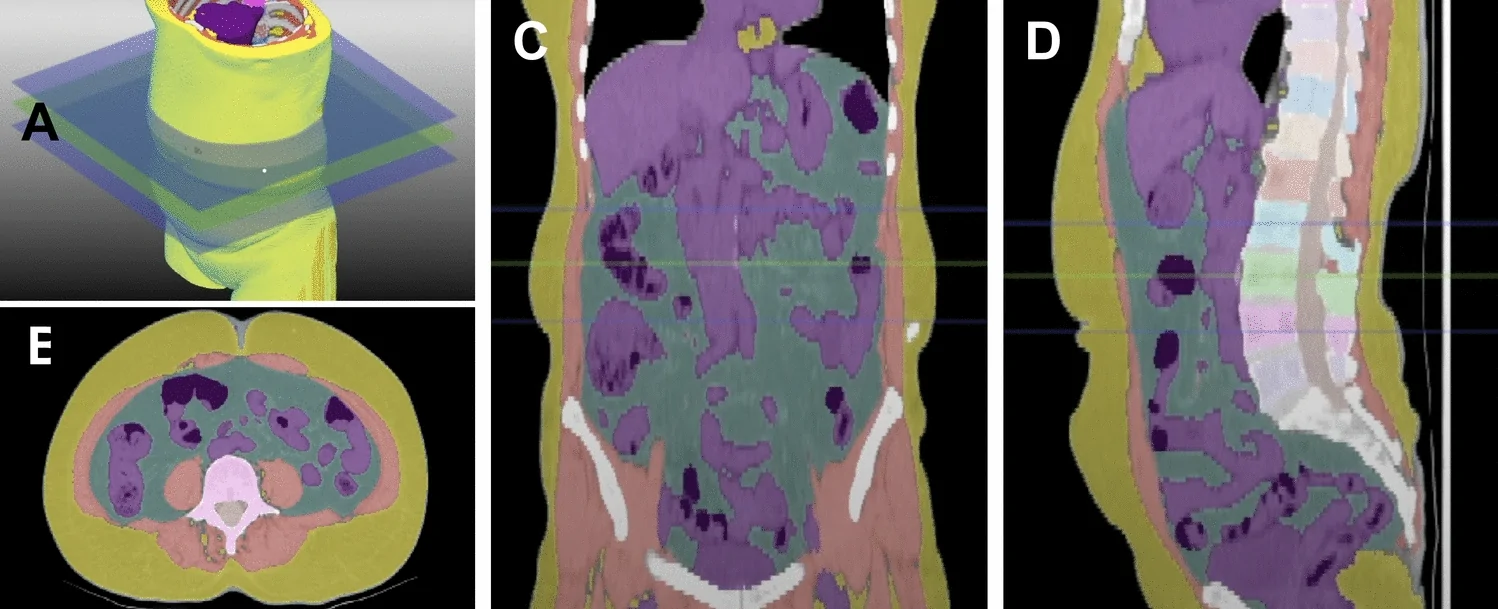
The workflow of AI-based automatic segmentation with volumetric (**A**), axial (**B**), coronal (**C**), and sagittal (**D**) representation

The abdominal–pelvic CT images of the enrolled patients were anonymized before being uploaded to a dedicated workstation. The anonymization protocol followed the internal Hospital protocol. The images were analyzed by using an automatic AI-based segmentation software (DeepCatch, v1.3.0.8486, MEDICAL IP Co. Ltd., Seoul, Republic of Korea) (Fig. 1), which performs an automatic segmentation of patients' abdominal CT scans with an accuracy of about 97% compared to a manual segmentation [15].

The software performed three different segmentations: (I) 2D segmentation: a single-slice segmentation of the abdominal CT scan at the level of the L3; (II) multislice 3D segmentation between the last rib and the superior iliac crest; and (III) a volumetric 3D segmentation including the entire abdominal volume, including all slices acquired without anatomical reference.

From these segmentations, body composition parameters were extracted, including volume (cm3), area (cm2), and average density, expressed in Hounsfield units (HU) of the following anatomical components: skin, bone, muscle, adipose visceral fat (AVF), subcutaneous fat (SF), internal organs (IO), and central nervous system (CNS), for both single-slice, multislices region, and whole volume. Muscle quality, bone density of the examined vertebrae, the diameter, and presence of calcifications of the abdominal aorta and a quantitative hepatic density analysis, steatosis (PDFF) were analyzed. Finally, by entering the weight and height of all patients, the skeletal muscle index (SMI) and the liver and spleen index (ratio, respectively, of the liver volume and the spleen volume to the height squared) were derived.

### Statistical analysis

Statistical analyses were performed with SPSS software (version 27.0; IBM Corp., Armonk, NY, USA). Body composition parameters were expressed as mean ± standard deviation. According to Gaussian normality or not, the comparisons were performed with the Student's t‐test and the Mann–Whitney U-test, respectively. All categorical variables were expressed with numbers and percentages. The body composition parameters were significantly compared between the two groups (Group 1 vs. Group 2), and the significant parameters were also tested with the receiver operating curve (ROC) to express the area under the curve (AUC), sensitivity, specificity, and cut‐off values, having the patient CRC-related death as the main endpoint.

To analyze the correlation between CRC-related death and body composition parameters, the Cox proportional hazards regression analysis was performed. Multivariable models were constructed including age, adjuvant chemotherapy, and pathological T and N stage as covariates. Hazard ratios (HRs) and 95% confidence intervals (CIs) were calculated. The parameters resulting in independent predictors of CRC-related death (HR≠1, *P* < 0.05) were selected for the survival analysis by using Kaplan–Meier curves with log-rank test, considering the overall survival as the time. *P*-values < 0.05 were considered significant.

## Results

### Study population

**Fig. 2:**
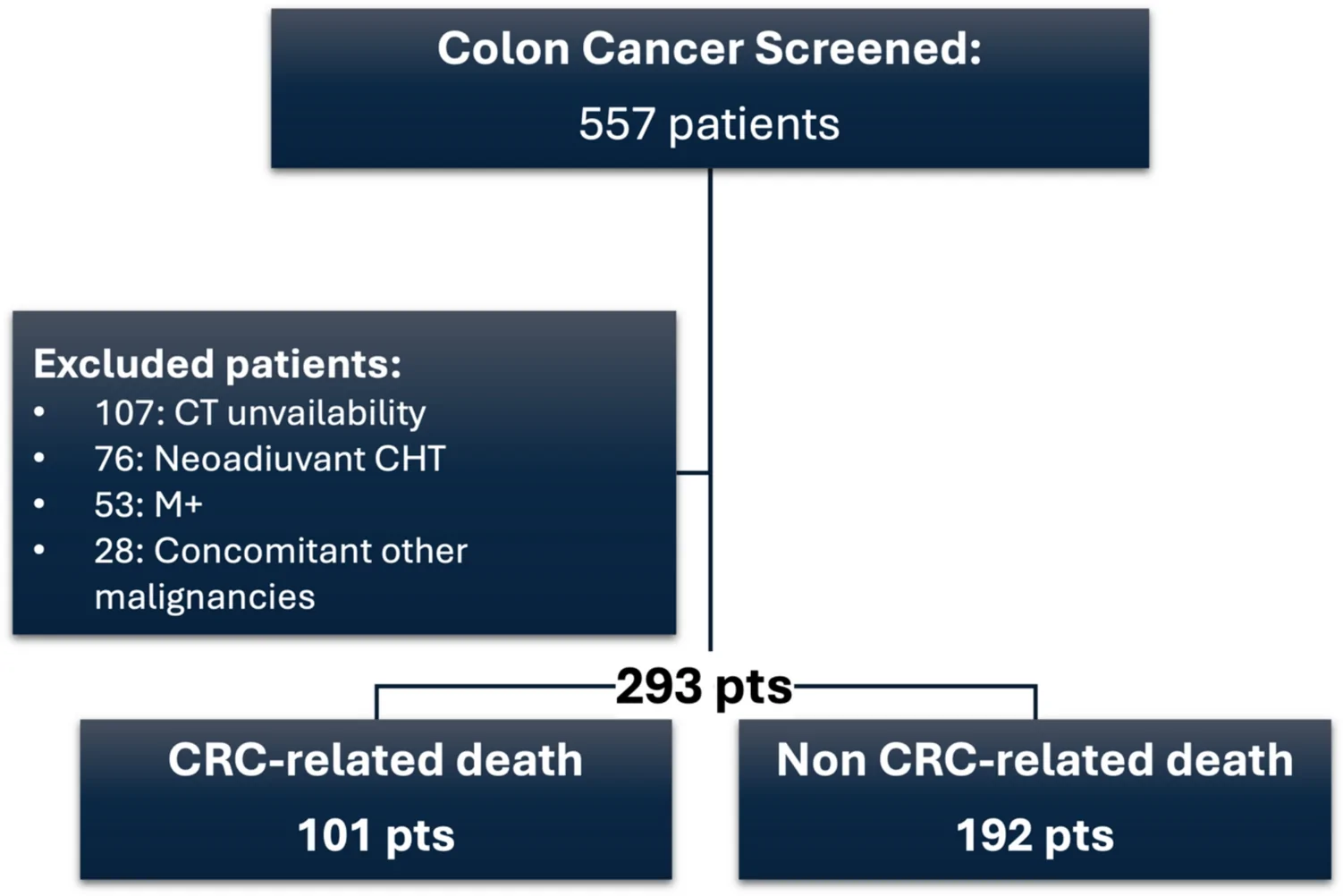
The flowchart of patient recruitment

According to the inclusion and exclusion criteria of our study, 293 patients affected by colon cancer at stages II–III were enrolled. For all patients, data regarding colon cancer-related mortality were collected, and 101/293 (34.5%) CRC-related deaths were identified from the Hospital death registry. In the CRC-related death sub-analysis, 101 patients had colon cancer-related death and were classified into Group 1 (CRC-related death), 192 patients were still alive or had died from causes unrelated to cancer, then they were classified into Group 2 (non-CRC-related death) (Fig. 2). Clinical data analysis showed that in Group 1, 57/101 (56.4%) were male (median age 76.5 years). In Group 2, 105/192 (54.6%) patients were male (median age of 68 years). Concerning adjuvant chemotherapy, 20/101 (19.8%) patients underwent adjuvant chemotherapy in Group 1 and 83/192 (43.2%) in Group 2.

Due to the retrospective nature of the study, BMI was calculated only for a subgroup of population (84/293, 28.7%), of which 28/84 (33.3%) with CRC-related death (Group 1a) and 54/84 (66.7%) non-CRC-related death (Group 2a). In Group 1a, the BMI was 26.13 kg/m^2^, and in the Group 2a, the BMI was 26.19 kg/m^2^ (*P* = 0.96).

From pathological staging, in Group 1, 11/101 (1%) patients were graded as pT1, 10/101 (9.9%) patients were considered as pT2, 57/101 (56.4%) patients were pT3, 22/101 (21.8%) patients were pT4a, 11/101 (10.9%) patients were graded as pT4b, 62/101 (61.4%) patients were pN0, and 39/101 (38.6%) patients were pN + . While in Group 2, 5/192 (2.6%) patients were pT1, 27/192 (14.1%) patients were pT2, 109/192 (56.8%) patients were pT3, 37/192 (19.2%) were pT4a, and 14/192 (7.3%) patients were pT4b; moreover, 134/192 (69.8%) patients were pN0, and 58/192 (30.2%) were pN + . Full results are listed in Table 1.

**Table 1:** Clinical and cancer-specific characteristics

	***N***	**%**
Group 1	101/293	34.5
Group 2	192/293	65.5
Group 1		
Male	57/101	56.4
Age (avg)	76.5	-
Adjuvant CHT	20/101	19.8
pTN		
T1	1/101	1
T2	10/101	9.9
T3	57/101	56.4
T4a	22/101	21.8
T4b	11/101	10.9
N +	39/101	38.6
Group 2		
Male	105/192	54.6
Age (avg)	68	-
Adjuvant CHT	83/192	43.2
pTN		
T1	5/192	2.6
T2	27/192	14.1
T3	109/192	56.8
T4a	37/192	19.2
T4b	14/192	7.3
N +	58/192	30.2

*CHT* Chemotherapy, *pTN* Pathological T and N staging

### Association between body composition and survival

The comparison between Group 1 and Group 2 in the multislice analysis and in the volume analysis showed that MeanHU AVF resulted in a statistically significant difference (all *P* < 0.05). The same parameter resulted in being significant in ROC analysis in both multislice and volume analysis, with an AUC of 0.61 (*P* < 0.05). The remanent parameters from volume, multislice, muscle, cardiovascular, and liver analysis, despite the statistically significant result, achieved AUC < 0.05 indicating performance well below chance level (AUC = 0.50), and suggesting a consistent tendency toward incorrect classification. The most clinically relevant parameters are reported in Tables 2 and 3.

**Table 2:** The performance in predicting CRC-related death of the most clinically relevant parameters extracted from multislice and volume analysis using the ROC (Receiver Operating Characteristic) curve

Parameters	Cutoff	*P*	ROC	CI
*Multislice analysis*				
MeanHU				
AVF				
ROC	> − 77.7 HU	0.002	0.61	0.54–0.68
SF				
ROC	< − 87.5 HU	0.212	0.54	0.48–0.61
*Volume analysis*				
MeanHU				
AVF				
ROC	> − 74.5 HU	0.003	0.61	0.53–0.7
SF				
ROC	> 81.5 HU	.028	0.58	0.51–0.65

AVF Adipose visceral fat, SF Subcutaneous fat, HU Hounsfield units

**Table 3:** The correlation analysis between body composition parameters and the CRC-related death

Parameters	Mean (IQR)	*P*	CI	HR (CI)
*Multislice analysis*				
MeanHU				
AVF				
Group 1 Group 2	− 77 HU (− 87; − 67) − 82 HU (− 90; − 76)	0.004	− 74.15; − 79.7 HU − 79.9; − 83.4 HU	1.03 (1.01–1.07)
SF				
Group 1 Group 2	− 87 HU (− 95; − 81) − 90 HU (− 98; − 82)	0.002	− 84.3; − 89.6 HU − 88.6; − 91.9 HU	1.04 (1.021–1.09)
Volume Analysis				
MeanHU				
AVF				
Group 1 Group 2	− 75 HU (− 85; − 65) − 80 HU (− 89; − 74)	0.04	− 72.4; − 77.8 HU − 78.3; − 81.63 HU	1.02 (0.99–1.03)

*AVF* Adipose visceral fat, *SF* Subcutaneous fat, *HU* Hounsfield Units

At univariable analysis, MeanHU of AVF and SF was associated with CRC-related death for the multislice analysis. In particular, lower attenuation values were observed in Group 1 compared to Group 2 for AVF (Group 1 vs. Group 2, − 77HU vs. − 82HU) and SF (Group 1 vs. Group 2, − 87HU vs − 90HU), and both parameters resulted to be directly correlated (*P* = 0.004 and HR = 1.03, *P* = 0.002 and HR = 1.04, respectively) with CRC-related death. Moreover, only MeanHU of AVF (Group 1 vs. Group 2, − 75HU vs. − 80HU) resulted to be directly correlated with CRC-related deaths also for the volumetric analysis (*P* = 0.04 and HR = 1.02) (Table 3).

In multivariable Cox regression analysis adjusted for age, adjuvant therapy, pathological T stage, and pathological N stage, visceral adipose tissue attenuation remained an independent predictor of CRC-related death in both multislice (HR 1.02, 95% CI 1.006–1.038, *P* = 0.007) and volumetric analysis (HR 1.021, 95% CI 1.005–1.038, *P* = 0.011). Subcutaneous adipose tissue attenuation remained significant only in the multislice analysis (HR 1.022, 95% CI 1.005–1.040, *P* = 0.012), but not in the volumetric model (*P* = 0.933), suggesting a less robust prognostic role.

The remanent parameters from volume, multislice, muscle, cardiovascular, and liver analysis did not demonstrate statistically significant differences.

Among the indexed parameters, there were no statistically significant results among BMI, muscle analysis (AVF index, SF index, total fat index, weight-to-muscle ratio, and skeletal muscle index), and liver analysis (liver index and spleen index) for both ROC curves and Cox regression analysis (*P* > 0.05).

### Survival analysis

The median of follow-up was 36 months after diagnosis. In Group 1, the median OS was 23 months, 24/101 (23.8%) patients had progression disease during follow-up with a median PFS of 12 months. In Group 2, 23/192 (12%) patients had progression during follow-up with a median PFS of 13 months.

**Fig. 3:**
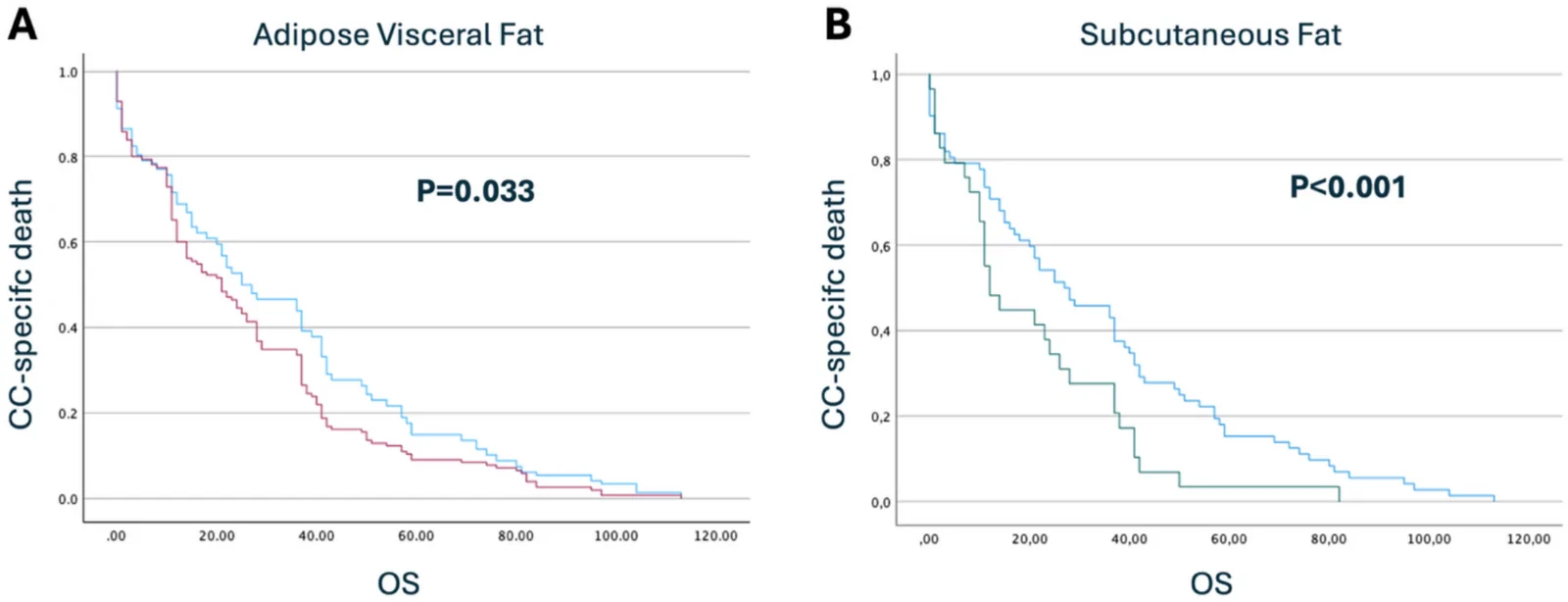
Survival analysis of MeanHU adipose visceral fat (**A**) and subcutaneous fat (**B**) for the multislice analysis by using Kaplan–Meier curves

In the Kaplan–Meier analysis with the log-rank test, the AVF and SF for the multislice analysis resulted to be statically significant (*P* = 0.033 and < 0.001, Chi-square = 4.56 and 11.7, respectively) (Fig. 3). While no significant results were achieved in the Kaplan–Meier analysis for the volumetric parameters. The indexed parameters, there were no statistically significant results among BMI, muscle analysis (AVF index, SF index, total fat index, weight-to-muscle ratio, and skeletal muscle index), and also for the liver analysis (liver index and spleen index).

## Discussion

Our study investigated the role of body composition parameters, analyzing the preoperative CT scans in non-metastatic colon cancer patients (stages II–III), as potential predictors of cancer-related death (CRC-related death). Using an AI-based volumetric segmentation tool, we assessed multiple body compartments and tissues including muscle, bone, visceral, and subcutaneous adipose tissue (AVF and SF), in both qualitative and quantitative (volume, area) terms. Our findings suggested that MeanHU AVF and SF, meant as density parameters, could be considered as predictors of CRC-related mortality. While indexed parameters, such as BMI or SMI, failed to show prognostic relevance. One of the most robust findings of our analysis was the significant correlation between higher AVF and SF density in cancer-related mortality group (Group 1). Although the ROC analysis demonstrated only moderate discriminatory ability (AUC ~ 0.61), these results should be interpreted with caution and not overemphasized. In our study, ROC curves were intended as a complementary measure of performance rather than the primary basis for inference. The main evidence supporting the prognostic role of adipose tissue density derives from the proportional hazards regression analysis, which evaluates time-to-event outcomes and provides a more appropriate framework for assessing independent associations. Therefore, the moderate AUC values do not contradict our primary findings but rather suggest that adipose tissue density alone may have limited discriminative power and could benefit from integration with additional clinical variables in future predictive models.

These findings were in line with recent literature, suggesting that adipose tissue density, reflecting underlying fat composition, metabolic activity, and inflammation, could serve as a qualitative biomarker of adipose tissue impairment [9, 12, 16]. Diversely from the classical point in which increased fat volume is associated with worse outcomes, recent literature has shown that adipose tissue’s higher density is associated with reduced lipid content and fibrosis, indicating pro-inflammatory changes [12]. In particular, Feliciano et al. [12] demonstrated that both in subcutaneous and visceral fat depots, a higher density was associated with increased mortality in colorectal cancer patients, independently from BMI and body weight changes, suggesting an impactful role of adipose tissue quality over adipose tissue quantity. Our study is in line with these findings, extending the cohort to patients with localized colorectal cancer.

Similarly, Furberg et al. [16] demonstrated that a higher visceral adipose tissue density, indicating prevalently lipid depletion and inflammation, was independently associated with both higher grade and advanced stage clear cell renal carcinoma. Also, Ebady et al. [17] demonstrated that HCC patients treated with selective internal radiation therapy and high visceral adipose tissue density had a two times higher risk of mortality in comparison with their counterpart with lower visceral adipose tissue density; these patients showed interestingly more prevalent portal hypertension clinical features and severe adverse events.

However, compared to the previous literature, our work adds several novel aspects; firstly, we evaluated an homogeneous cohort of non-metastatic stages II–III colon cancer patients, whereas the previous studies mainly included mixed stage or metastatic disease patients. Moreover, we evaluated cancer-related death as a primary endpoint, rather than overall survival. Furthermore, higher visceral adipose tissue density has been observed to be a significant biomarker for metabolic dysfunction and associated with higher cardiometabolic risk and adverse biomarkers, such as higher insulin resistance and blood pressure, and higher adipokine levels and lower high-density lipoprotein levels [16].

Moreover, our study was based on the evaluation of body composition parameters using an AI-driven software for CT segmentation, which allowed a reproducible, automated, and comprehensive extraction of the features. Overcoming the 2D single-slice segmentation which supported most of the studies in this field [18–20], by shortening analysis time and reducing inter-observer variability [21]. As preoperative CT is a standardized method in colon cancer workup, a potential involvement of an automated AI-based body composition evaluation tool would be easily integrated in clinical practice, without needing additional testing. Our evaluation was based on three segmentations approaches: 2D L3 single-slice, multislice approach, from the last rib to the iliac crest, and whole abdominal volume evaluation. Among these different approaches, the multislice evaluation emerged as the most performant, with both AVF and SF density showing significant associations with survival in ROC and Cox regression analysis*.* The findings suggest that a multislice targeted body composition approach, as of subcutaneous and visceral fat evaluation, could be more relevant clinically. Furthermore, as demonstrated by Lim et al. [22] and Kim et al. [23], full-volume and 2D analysis could overestimate or underestimate compartments, eventually adding prognostic value to AI-based body composition analysis.

Despite the previous literature confirming the role of sarcopenia as a prognostic biomarker in oncologic patients [24], our study did not confirm this association. In fact, SMI and muscle-related indices did not significantly differ between groups both in univariate and multivariate analyses. This discrepancy could be partially due to the nature of the population, in fact our study included only non-metastatic stages II–III colon cancer patients not receiving neoadjuvant therapy. In this setting, muscle loss frequently observed in advanced cancer stages or during chemotherapy, might be less evident, eventually reducing sarcopenia prognostic impact.

Also, muscle quality could be considered as a more sensitive biomarker than muscle mass, an aspect which was not explored in our study, necessitating for a more in-depth evaluation and further investigations. Our findings also suggested the limited utility of BMI as a prognostic tool, as both BMI- and non-BMI-related indices did not correlate significantly with survival; this finding is consistent with various literature suggesting BMI lack of sensitivity to discriminate between fat and lean mass, not reflecting the biological activity of body tissues [25].

One of the most significant findings of this study suggested that adipose tissue’s density correlated with cancer-related death, even when considering pathological staging. This result was found significant in survival analyses, strongly supporting the hypothesis that adipose tissue’s density could be associated with reduced treatment response or a more aggressive disease. As adipose tissue density values could be automatically extracted from routinary follow-up CT scans, adding these metrics to evaluate preoperative risk stratification could be easily integrated in clinical practice; moreover, adipose tissue HU values could be considered to tailor personalized therapy regimens, including nutritional or metabolic interventions aimed at improving adipose tissue health.

Furthermore, the overall predictive power of body composition parameters was found significant, potentially suggesting that multimodal approaches including body composition, molecular tumor markers, and inflammatory biomarkers could be more effective in prognostic prediction.

Even if the results are promising, there are some limitations to the study that need to be addressed. Firstly, the retrospective nature of the study and the fact that we included a relatively small population, considering colon cancer prevalence. Secondly, BMI data were available in less than 30% of patients, limiting the reliability of comparisons with CT-derived biomarkers and warranting caution in interpreting its lack of prognostic significance. Also, we demonstrated a potential role of density in mortality prediction but did not assess its relationship with surgical complications or recurrence rate. Furthermore, we provided a single body composition software evaluation and did not acknowledge the potential inter-scanners variability. In addition, clinically meaningful HU thresholds were not defined, limiting the immediate applicability of these findings in clinical practice and warranting further investigation in larger, prospective cohorts. One more potential limitation of our study was the large number of body composition parameters explored, which may increase the risk of type I error. However, these analyses were partly exploratory, and many of the evaluated variables are highly correlated, making strict multiple comparison corrections (e.g., Bonferroni) potentially overconservative. Moreover, the primary inferential evidence of our study is based on Cox regression models rather than multiple independent hypothesis tests. Nonetheless, this aspect should be considered when interpreting the results, and further validation in independent cohorts is warranted.

## Conclusions

In conclusion, our study demonstrated that higher visceral and subcutaneous fat density in preoperative CT scans was significantly associated with increased rates of cancer-related mortality in non-metastatic colon cancer patients. The findings suggest that a targeted, qualitative evaluation of body composition parameters, including adipose tissue density, could potentially serve as independent biomarkers of prognostic outcomes. Using an AI-based volumetric body composition analysis tool may enhance the reproducibility and feasibility of body composition assessment, providing a valid tool for risk stratification. However, the biomarkers’ performance was moderate (AUC = 0.61), indicating that this imaging biomarker is not yet completely clinically actionable; our results should be supported by further validation in larger, prospective cohorts.

Future directions include evaluation of the biological mechanisms linking adipose tissue HU values to cancer progression, including tissue inflammation or metabolic dysfunctions. Additionally, studies targeting body composition through interventional approaches as exercise, diet therapy of pharmacological modulation, could help determine the potential outcomes improvement in colon cancer patients by modifying adipose tissue quality.
